# Association between gestational age at birth, antenatal corticosteroids, and outcomes at 5 years: multiple courses of antenatal corticosteroids for preterm birth study at 5 years of age (MACS-5)

**DOI:** 10.1186/1471-2393-14-272

**Published:** 2014-08-13

**Authors:** Elizabeth Asztalos, Andrew Willan, Kellie Murphy, Stephen Matthews, Arne Ohlsson, Saroj Saigal, Anthony Armson, Edmond Kelly, Marie-France Delisle, Amiram Gafni, Shoo Lee, Renee Sananes, Joanne Rovet, Patricia Guselle, Kofi Amankwah, for the MACS-5 Collaborative Group

**Affiliations:** Department of Newborn & Developmental Paediatrics, Sunnybrook Health Sciences Centre, Sunnybrook Research Institute, University of Toronto, Toronto, Ontario Canada; Program in Child Health Evaluative Sciences, SickKids Research Institute, Dalla Lana School of Public Health, University of Toronto, Toronto, Ontario Canada; Department of Obstetrics and Gynaecology, Mount Sinai Hospital, University of Toronto, Toronto, Ontario Canada; Departments of Physiology, Obstetrics and Gynecology and Medicine, University of Toronto, Toronto, Ontario Canada; Department of Paediatrics, Mount Sinai Hospital, University of Toronto, Toronto, Ontario Canada; Department of Pediatrics, McMaster University Medical Centre, Hamilton, Ontario Canada; Department of Obstetrics and Gynecology, IWK Health Centre, Dalhousie University, Halifax, Nova Scotia Canada; Department of Obstetrics and Gynecology, BC Women’s Hospital, University of British Columbia, Vancouver, British Columbia Canada; Centre for Health Economics and Policy Analysis, Department of Clinical Epidemiology and Biostatistics, McMaster University, Hamilton, Ontario Canada; Department of Psychology, The Hospital for Sick Children, University of Toronto, Toronto, Ontario Canada; Neuroscience and Mental Health Program, The Hospital for Sick Children, Department of Paediatrics, University of Toronto, Toronto, Ontario Canada; The Hospital for Sick Children, Toronto, Ontario Canada; Department of Obstetrics & Gynecology, Mackenzie Health, Richmond Hill, Ontario Canada; The Centre for Mother, Infant, and Child Research, Sunnybrook Research Institute, Sunnybrook Health Sciences Centre, M4-230, 2075 Bayview Ave., Toronto, M4N 3 M5Ontario, Canada

**Keywords:** Preterm birth, Long-term neurodevelopmental outcomes, Antenatal corticosteroids, Gestational age at birth

## Abstract

**Background:**

The Multiple Courses of Antenatal Corticosteroids for Preterm Birth Study (***MACS***) showed no benefit in the reduction of major neonatal mortality/morbidity or neurodevelopment at 2 and 5 years of age. Using the data from the randomized controlled trial and its follow-up, the aim of this study was to evaluate the association between gestational ages at birth in children exposed to single versus multiple courses of antenatal corticosteroid (ACS) therapy in utero and outcomes at 5 years of age.

**Method:**

A total of 1719 children, with the breakdown into groupings of <30, 30–36, and ≥ 37 weeks gestation at birth, contributed to the primary outcome: death or survival with a disability in one of the following domains: neuromotor, neurosensory, and neurobehavioral/emotional disability and were included in this analysis.

**Results:**

Gestational age at birth was strongly associated with the primary outcome, *p* < 0.001. Overall, the interaction between ACS groups and gestational age at birth was not significant, *p* = 0.064. Specifically, in the 2 preterm categories, there was no difference in the primary outcome between single vs. multiple ACS therapy. However, for infants born ≥37 weeks gestation, there was a statistically significant increase in the risk of the primary outcome in multiple ACS therapy, 48/213 (22.5%) compared to 38/249 (15.3%) in the single ACS therapy; OR = 1.69 [95% CI: 1.04, 2.77]; *p* = 0.037.

**Conclusion:**

Preterm birth (<37 weeks gestation) remained the primary factor contributing to an adverse outcome regardless of the number of courses of ACS therapy. Children born ≥ 37 weeks and exposed to multiple ACS therapy may have an increased risk of neurodevelopmental/neurosensory impairment by 5 years of age. To optimize outcomes for infants/children, efforts in reducing the incidence of preterm birth should remain the primary focus in perinatal research.

**Trial registration:**

This study has been registered at (identifier NCT00187382)

**Electronic supplementary material:**

The online version of this article (doi:10.1186/1471-2393-14-272) contains supplementary material, which is available to authorized users.

## Background

The Multiple Courses of Antenatal Corticosteroids for Preterm Birth Study (***MACS***) is an international, multicenter, double-masked, randomized controlled trial that compared multiple courses of antenatal corticosteroids (ACS) given every 14 days to a single course in women at increased risk for preterm birth [1]. The initial report found that infants born to women in the multiple ACS group had similar composite mortality and morbidity (severe respiratory distress syndrome, grade III or IV intraventricular hemorrhage, periventricular leukomalacia, bronchopulmonary dysplasia, or necrotizing enterocolitis) as compared with those in the single course group [1]. However, multiple courses of ACS reduced fetal growth. At 18–24 months of age, there were no significant differences in death or neurologic disability (cerebral palsy or cognitive delay) in children born to women in the multiple antenatal corticosteroids group compared to the placebo group [2]. At 5 years of age, no significant differences in the primary composite outcome of death or neurodevelopmental disability were seen between the two groups [3].

Using the data from the randomized controlled trial and its follow-up, the aim of this study was to perform a secondary analysis examining the association of gestational age at birth on the comparison between single and multiple courses of ACS therapy with respect to outcomes at 5 years of age.

## Methods

### Initial study and 18–24 months and 5-year follow-up

Women were enrolled in the ***MACS*** if they were between 25 and 32 weeks of gestation, remained pregnant 14 to 21 days after an initial course of antenatal corticosteroid therapy (either betamethasone or dexamethasone) therapy, and continued to be viewed as at high risk of preterm birth by their clinicians. Women were not eligible if they had a contraindication to corticosteroid use, needed chronic treatment with these drugs, demonstrated evidence of chorioamnionitis, carried a fetus with a known lethal congenital anomaly, had received an initial course of prenatal corticosteroid therapy before 23 weeks of gestation, or had participated previously in the ***MACS*** trial. In multiple-fetus pregnancies, if a fetus was thought to have died prior to 13 weeks, that fetus was not considered part of the pregnancy for the purposes of this study. Women assigned to the multiple courses arm received two doses of 12 mg betamethasone intramuscularly 24 hours apart; those assigned to the single course arm received a similarly appearing placebo injection. The study medication was given every two weeks until 33 weeks of gestation or birth, whichever occurred first. Randomization took place from April 9, 2001 to August 31, 2006.

All children alive at 5 years of age underwent the 5-year assessment which included a neurologic assessment to determine the presence of cerebral palsy and any hearing/visual difficulties, and the completion of two parent questionnaires. The 5-year study and all secondary analyses were approved by the Research Ethics Board at the Sunnybrook Health Sciences Centre. The institutions were encouraged to contact the families of all surviving children even if no contact had been made at 18–24 months of age for the 18–24 month follow-up assessment. The target date for the visit was the child’s 5th birthday; completing the assessments within 4 months of the target date was encouraged but efforts to locate and assess the children continued beyond this age when necessary. Following ethics approval at each institution, written informed consent was obtained from a parent or guardian. The 5-year follow-up began in June 2006 and was complete by May 2012.The primary outcome of the 5-year follow-up was a composite of death or survival with a neurodevelopmental disability in at least one of the following domains: neuromotor (non-ambulatory cerebral palsy), neurosensory (blindness, deafness or a need for visual or hearing aids), or neurocognitive/neurobehavioral (abnormal attention, memory or behaviour) function. Non-ambulatory cerebral palsy was present if the child had a non-progressive motor impairment characterized by abnormal muscle tone and decreased range of movements, with a gross motor function of 3–5 as defined in the Gross Motor Function Classification System (GMFCS) [4]. Neurosensory disability was defined as blindness, deafness, or need for visual or hearing aids based on local criteria. Neurocognitive/neurobehavioral disability was defined as an abnormally elevated score (>1.5standard deviations above the normative control sample) on either one of two parent-administered questionnaires: the Behavior Rating Inventory of Executive Function- Preschool version (BRIEF-P) and the Child Behavior Checklist-1½-5 (CBCL-1½-5) [5, 6].

### Analysis

The primary outcome and its components were analysed using a general linear model for a binary response (*i.e.* logistic regression) with repeated measures for children from the same pregnancy. The model included ACS treatment group (multiple, single), gestational age at birth group (<30, 30–36, ≥37 weeks), plus its interaction with ACS treatment group. The model included the stratification variable gestational age at randomization (26–27 vs. 28+ weeks) and, if the variables were significant at the two-sided level of 0.1, the following covariates from Table 1: preterm pre-labour rupture of membranes (yes/no), multiple pregnancy (yes/no), maternal smoking (yes/no), parity (0, 1+), sex of the infant and the country’s perinatal mortality rate (<=10/1000, >10-20/1000, >20/1000). Because there were very few neuromotor outcomes, the model would not converge and no results for this component of the primary outcome are given. Model estimation was facilitated by generalized estimating equations, using PROC GENMOD in SAS. Odds ratios plus 95% confidence intervals were determined. This research adhered to the STROBE guidelines for observational studies.

**Table 1:** **Characteristics at randomization into** ***MACS*** **by gestational age at delivery**

Characteristic	Preterm (<30 weeks) N = 158 women	Preterm (30–36 weeks) N = 781 women	Term (≥37 weeks) N = 437 women
Mean maternal age (years) (SD)	29.3 (6.3)	29.8 (6.2)	28.1 (6.2)
Treatment group			
Single course	76 (48.1%)	375 (48.0%)	236 (54.0%)
Repeat courses	82 (51.5%)	406 (52%)	201 (46.0%)
Multiple pregnancy	37 (23.4%)	244 (31.2%)	26 (5.9%)
Number of fetuses			
Singleton	121 (77%)	537(69%)	411 (94%)
Twin	29 (18%)	197 (25%)	25 (6%)
Triplet	8 (5%)	47 (6%)	1 (<0.01%)
Mean gestational age at randomization (weeks) (SD)	27.2 (1.1)	29.7 (1.9)	29.6 (1.9)
Gestational age at randomization (weeks)			
< 25	0 (0%)	0 (0%)	0 (0%)
25-27	114 (72%)	163 (21%)	91 (21%)
28-32	44 (28%)	618 (79%)	334 (79%)
> 32	0 (0%)	0 (0%)	2 (<0.01%)
Preterm pre-labor rupture of membranes at randomization	77 (49%)	138 (18%)	8 (1.8%)
National perinatal mortality rate of country^b^			
≤ 10/1000	127(80%)	553 (71%)	224 (51%)
> 10 - 20/1000	22 (14%)	187 (24%)	175 (40%)
> 20/1000	9 (6%)	41 (5%)	38 (9%)
Number of previous pregnancies			
0	50 (32%)	245 (31%)	103 (24%)
1-4	91 (57%)	465 (60%)	287 (66%)
>4	17 (11%)	70 (9%)	45 (10%)
Missing		1 (<0.01%)	2 (<0.01%)
Maternal smoking	15 (9%)	64 (8%)	62 (14%)
Number of courses of study drug			
0	2 (1.2%)	3 (<0.01%)	1 (<0.01%)
1	128(81%)	312 (40%)	109 (25%)
2	26 (16%)	225 (33%)	156 (36%)
3	1 (0.1%)	133 (17%)	103 (24%)
4	0 (0%)	78 (10%)	68 (16%)
Missing	1 (0.1%)	0 (0%)	0 (0%)

^a^1 child lost to follow-up case earlier in trial found and included at 5 years, no maternal data available.

^b^Countries with a national perinatal mortality rate of ≤10/1000 were: Canada, Chile, Denmark, Germany, Hungary, Israel, the Netherlands, Poland, Spain, Switzerland, United Kingdom, United States; countries with a national perinatal mortality rate of >10 - 20/1000 were: Argentina, Brazil, Peru; countries with a national perinatal mortality rate of >20/1000 were: Bolivia, China, Colombia, Jordan, Russia.

## Results

### Study participants

**Figure 1:**
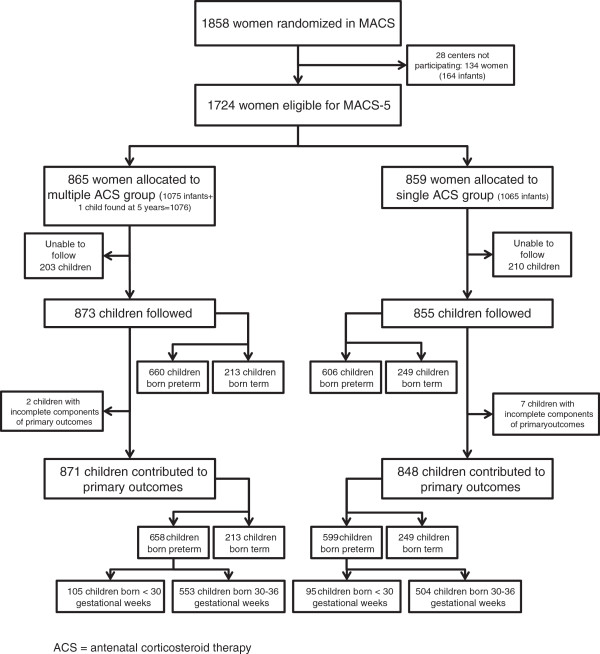
**Study profile.** This study profile outlines the recruitment of the children born preterm and term.

Figure 1 illustrates the study group for ***MACS-5***. For the main 5-year follow-up, 413 children were unable to be followed because they could not be located or parents declined participation, 1 child lost for the neonatal and 2 year follow-up phase was found, leaving 1728 children (80.7% of the 2141 eligible children) to contribute to the outcomes of the 5-year follow-up. Nine children had insufficient information to contribute to the primary outcome. In total, 1719 children contributed to the primary outcome of ***MACS-5*** and it is these children, broken down into groupings of <30, 30–36, and ≥ 37 weeks gestation at birth, who contribute to this secondary analysis. The median age for the 5 year assessments was 5.2 years.

The baseline maternal characteristics and co-interventions after randomization are outlined in Table 1 by gestational ages at birth. A summary of the neonatal, 18–24 months and 5-year neurodevelopmental outcomes of the study cohort are outlined in Table 2. The baseline neonatal outcomes of the infants followed compared to those not followed are outlined in the Additional file 1: Table S1.

**Table 2:** **Perinatal/neonatal, 18–24 months, and 5-year outcomes of infants/children by gestational age at delivery**

Outcomes	Preterm (<30 weeks) N = 200	Preterm (30–36 weeks) N = 1066	Term (≥37 weeks) N = 462 ^a^
Gender^b^			
Male	106 (53%)	560 (53%)	240 (52%)
Female	94 (47%)	506 (47%)	222 (48%)
Death or serious neonatal morbidity: composite primary outcome (one or more of death, severe RDS^c^, BPD^d^, IVH^e^ [grade III/IV], cystic PVL^f^, NEC^g^)	126 (64%)	107 (10%)	2 (<0.01%)
Missing	0 (0%)	0 (0%)	1 (<0.01%)
Death or neurologic impairment at 18–24 months: composite primary outcome (one or more of death, cerebral palsy or cognitive delay)	89 (45%)	128 (12.0%)	27 (6%)
Missing	1 (0.01%)	5 (0.1%)	2 (<0.01%)
Death or severe disability (neuromotor, neurosensory, neurocognitive) at 5 years §	104 (52%)	237 (22%)	86 (19%)

^a^1 lost to follow-up case earlier in trial found and included at 5 years; no neonatal and 18–24 months data available;

^b^New information found on gender.

^c^RDS = respiratory distress syndrome; ^d^BPD = bronchopulmonary dysplasia;

^e^IVH = intraventricular hemorrhage; ^f^PVL = periventricular leukomalacia;

^g^NEC = necrotising enterocolitis.

§Clinical information of 19 cases missing/inadequate questionnaires were reviewed by an adjudication committee (10 cases were adjudicated to contribute to analysis and primary outcome; 9 had insufficient information).

### Outcome

The primary outcome and its components at 5 years of age are given in Table 3 by ACS treatment groups and gestational age at birth. The covariate results for the primary outcome and its components are given in Table 4, and include the odds ratios, confidence intervals and levels of significance. The odds ratios, confidence limits and significance levels for the primary outcome and its components are given by gestational age at birth in Tables 5 and 6; the results were similar when the covariates were omitted.

**Table 3:** **Outcomes of children at 5 years by gestational age at birth and ACS groupings**

	Preterm (<30 weeks) N = 200		Preterm (30–36 weeks) N = 1066		Term (≥37 weeks) N = 462	
Outcome	Multiple ACS N = 105	Single ACS N = 95	Multiple ACS N = 553	Single ACS N = 504	Multiple ACS N = 213 ^a^	Single ACS N = 249
Death or severe disability (neuromotor, neurosensory, neurocognitive)§	54/105 (51.4%)	50/95 (52.6%)	115/553 (20.8%)	122/504 (24.2%)	48/213 (22.5%)	38/249 (15.3%)
Death up to 5 years	29/105 (27.6%)	23/95 (24.2%)	16/555 (2.9%)	22/511 (4.3%)	1/213 (0.5%)	2/249 (0.8%)
Neuromotor disability †† (non-ambulatory cerebral palsy)	1/76 (1.3%)	6/72 (8.3%)	3/539 (0.6%)	4/489 (0.8%)	0/212 (0%)	1/247 (0.4%)
Neurosensory disability	13/76 (17.1%)	13/72 (18.1%)	38/539 (7.1%)	41/489 (8.4%)	19/212 (9.0%)	7/247 (2.8%)
Needing visual aids	9/76 (11.8%)	11/72 (15.3%)	35/539 (6.5%)	34/489 (7.0%)	17/212 (8.0%)	7/247 (2.8%)
Deafness	4/76 (5.3%)	2/72 (2.8%)	4/539 (0.7%)	3/489 (0.6%)	3/212 (1.4%)	1/247 (0.4%)
Pre-existing (diagnosed at 2 years or earlier)	3	2	1	2	0	0
New (diagnosed after 2 years of age)	1	0	3	1	3	1
Neurocognitive/neurobehavioural disability	15/76 (19.7%)	16/69 (23.2%)	62/534 (11.6%)	63/478 (13.2%)	31/212 (14.6%)	30/246 (12.2%)

ACS= antenatal corticosteroid.

^a^1 child lost to follow-up earlier in trial found and included at 5 years.

§Clinical information of 19 cases missing/inadequate questionnaires were reviewed by an adjudication committee (10 cases were adjudicated to contribute to analysis and primary outcome; 9 had insufficient information).

††non-ambulatory cerebral palsy was defined as Gross Motor Function Classification System III, IV or V.

**Table 4:** **Covariates associated with neurodevelopmental outcomes at 5 years of age**

Death or severe disability			
Covariate	Comparison	OR [ 95% confidence limits]	***p*** -value
Gestational age at randomization	26-27 vs. 28+ weeks	1.02 [0.76, 1.38]	0.897
Premature rupture of membranes	Yes vs. No	1.58 [1.14, 2.18]	0.009
Mother smokes	Yes vs. No	1.62 [1.10, 2.40]	0.023
Parity	1+ vs. 0	1.26 [1.02, 1.56]	0.033
Country PMR^a^	>10/1000 vs. ≤10/1000	1.33 [1.09, 1.61]	0.006
**Neurosensory disability**			
**Covariate**	**Comparison**	**OR [ 95% confidence limits]**	***p*** **-value**
Gestational age at randomization	26-27 vs. 28+ weeks	1.04 [0.67, 1.60]	0.877
Premature rupture of membranes	Yes vs. No	1.58 [0.94, 2.64]	0.110
**Neurocognitive**			
Gestational age at randomization	26-27 vs. 28+ weeks	0.94 [0.64, 1.40]	0.764
Multiple pregnancy	Yes vs. No	0.60 [0.41, 0.89]	0.006
Maternal smoking	Yes vs. No	2.18 [1.44, 3.31]	0.002
Sex of the baby	Male vs. Female	1.65 [1.23, 2.20]	0.001
Country PMR^a^	>10/1000 vs. ≤10/1000	1.40 [1.10, 1.78]	0.012
**Death**			
Gestational age at randomization	26-27 vs. 28+ weeks	1.42 [0.84, 2.40]	0.1984
Premature rupture of membranes	Yes vs. No	2.08 [1.27, 3.41]	0.0089
parity	1+ vs. 0	1.44 [0.98, 2.11]	0.0700
Country PMR^a^	>10/1000 vs. ≤10/1000	2.37 [1.69, 3.33]	0.0001

^a^PMR = perinatal mortality rate.

Countries with a national perinatal mortality rate of ≤10/1000 were: Canada, Chile, Denmark, Germany, Hungary, Israel, the Netherlands, Poland, Spain, Switzerland, United Kingdom, United States; countries with a national perinatal mortality rate of >10 - 20/1000 were: Argentina, Brazil, Peru; countries with a national perinatal mortality rate of >20/1000 were: Bolivia, China, Colombia, Jordan, Russia.

**Table 5:** **Adjusted odds ratios, confidence limits and** ***p*** **-values by gestational age at birth**

Outcome	Gestational age at birth	OR [ 95% confidence limits]	***p*** -value	***p*** -value for interaction
Death or severe disability^a^	< 30 weeks	0.85 [0.35, 2.11]	0.608	0.064
	30-36 weeks	0.84 [0.52, 1.36]	0.262	
	**≥ 37 weeks**	**1.69 [1.04, 2.77]**	0.037*	
Neurosensory disability^b^	< 30 weeks	0.85 [0.35, 2.11]	0.732	0.021
	30-36 weeks	0.84 [0.52, 1.36]	0.481	
	**≥ 37 weeks**	**3.70 [1.57, 8.75]**	0.004*	
Neurocognitive^c^	< 30 weeks	0.84 [0.35, 2.01]	0.698	0.605
	30-36 weeks	0.91 [0.61, 1.36]	0.632	
	≥ 37 weeks	1.31 [0.75, 2.29]	0.347	
Death^d^	< 30 weeks	1.10 [0.54, 2.24]	0.794	0.587
	30-36 weeks	0.68 [0.35, 1.31]	0.247	
	≥ 37 weeks	sparse data, no convergence		

^a^adjusted for gestational age at randomization, premature rupture of membranes, maternal smoking, parity and the country’s perinatal mortality rate.

^b^adjusted for gestational age at randomization and premature rupture of membranes.

^c^adjusted for gestational age at randomization, multiple pregnancy, maternal smoking, sex of the baby and the country’s perinatal mortality rate.

^d^adjusted for gestational age at randomization, premature rupture of membranes, parity and the country’s perinatal mortality rate.

**Table 6:** **Unadjusted odds ratios by gestational age at birth**

Outcome	Gestational age at birth	OR
Death or severe disability	< 30 weeks	0.92
	30-36 weeks	0.83
	≥ 37 weeks	1.64
Neurosensory disability	< 30 weeks	0.90
	30-36 weeks	0.81
	≥ 37 weeks	3.48
Neurocognitive	< 30 weeks	0.76
	30-36 weeks	0.90
	≥ 37 weeks	1.30
Death	< 30 weeks	1.19
	30-36 weeks	0.66
	≥ 37 weeks	0.58

Gestational age at birth was strongly associated with primary outcome as defined for 5 years of age, *p <*0.001. The interaction between ACS group and gestational age at birth was not significant, *p* =0.064 (Table 5). In the two preterm groups, there was no difference seen in the primary outcome or its components between ACS groups. However, in children born at term, there was a statistically significant increase in the risk of the primary outcome in the multiple ACS group compared to the single ACS group, 48/213 (22.5%) vs. 38/249 (15.3%); OR = 1.69 [95% CI: 1.04, 2.77]; *p* = 0.037 (Table 5). In addition, there was a significant interaction between ACS group and gestational age at birth for neurosensory disability (*p* = 0.021), and for the children born at term there was a significant increase in the risk of neurosensory disability in the multiple ACS group: 19/212 (9.0%) vs. 7/247 (2.8%); OR = 3.70 [95% CI: 1.57, 8.75]; *p* = 0.004. There was no interaction between ACS group and gestational age at birth with respect to neurocognitive/neurobehavioral disability or death.

## Discussion

In this secondary analysis, we were able to demonstrate that preterm birth remained the primary driver contributing to an adverse neurodevelopmental outcome regardless of the number of courses of ACS. The earlier an infant was born, the higher the chances of experiencing an adverse outcome. In this study, half of the children who were born <30 weeks gestation were presenting with at least one of the components of the composite outcome. These figures are consistent with many studies that show that the more preterm infant continues to be at high-risk for adverse outcomes and that these numbers have not changed dramatically over the past decades [7–10]. Children born between 30–36 weeks gestation were found to still have a significant risk of an adverse outcome but not as high as the more preterm group.

As with most interventions there are benefits but also potential risks that must be balanced. ***MACS*** found no improvement in preterm birth outcomes and demonstrated a significant decrease in birth weight, length and head circumference following administration of multiple ACS, generating concern that there may be potential for harm [1]. With the clinical diagnosis of preterm labor being imprecise and interventions directed to preventing or delaying preterm birth implemented, at least a third of women can go on to give birth at term [11]. Previous studies which have evaluated the relationship of any effects of ACS on the infants are often complicated by the fact that most of the infants in these studies are preterm and are already at risk for delayed growth and development.

In ***MACS***, 32% of the women gave birth at term [1]. Infants born at term are unlikely to benefit from multiple courses of ACS as they are not required for pulmonary maturation. In the ***MACS*** cohort, less than 1% of the infants born at term had any components of the primary neonatal outcomes and less than 5% of the adverse neurodevelopmental outcomes at 18–24 months [2]. Yet the children exposed to multiple courses of ACS as fetuses and had gone on to be born at term had an almost 4-fold increased odds of neurosensory difficulties by 5 years of age. We also previously showed that this risk of difficulties was not dose-dependent with ACS therapy [3]. Although the absolute numbers for these impairments are small, these differences may have important clinical implications.

The term infant may be seen as one means of observing specific effects of ACS as they, in general, are less at risk for many of the growth and neurologic impairments often seen in children born preterm [12, 13]. More recently, children born at term have been studied to evaluate the potential long-term effects of ACS. Davis et al. noted that fetal exposure to ACS had neurologic consequences that persisted for at least 6 to 10 years as manifested by thinner cortex measurements on MRI [12], with the regions most affected being those involved with affective disorders suggesting increased vulnerability to mental health issues. Similarly, Alexander et al. showed that there were long-lasting effects of ACS exposure on hypothalamic-pituitary-adrenal reactivity in term-born children which have implications regarding the vulnerability for health and mental disorders [14].

Animal studies have demonstrated the deleterious effects of multiple courses of ACS on critical nerves within the central nervous system such as the optic and auditory nerve [12–14]. Dunlop and Quinlivan examined the effect of repeated injections of corticosteroids on the development of the optic nerve and reported a disrupted retinal development that persisited days after cessation of treatment and a significant delay in the myelination of the optic axons and thinning of the retina [15, 16]. Church et al. demonstrated that neonatal rats exhibited prolonged auditory brainstem response latencies reflecting slower neural transmission times along the auditory nerve and brainstem auditory pathway, and associated hearing deficits [17]. Similar effects may be present to account for the differences seen in the term group of this study.

There are limitations to this secondary analysis, as the groups based on gestational age at birth were defined after randomization thus making it plausible that there may have been pre-existing differences to account for the findings. Notwithstanding, the strengths of the study are that the data came from a large randomized controlled trial and that greater than 80% of the cohort has been followed. The neonatal outcomes of the children who were followed and those who were not followed were comparable reassuring us that there was a balance in the gestational groups and that the numbers were not inappropriately inflated in one group more so than another. Thus the results do present some concern about potential long-term consequences of multiple ACS therapy which clinicians need to consider especially if the benefits of multiple ACS are not substantial.

## Conclusions

In this secondary analysis, preterm birth was found to be the primary factor contributing to an adverse neurodevelopmental outcome regardless of the number of courses of ACS. Children born ≥ 37 weeks and exposed to multiple ACS therapy may have an increased risk of neurodevelopmental/neurosensory impairment. To optimize outcomes for infants/children, efforts in reducing the incidence of preterm birth should remain a primary focus in perinatal research as the morbidities associated with preterm birth continue to play an important role in the presence of long-term outcomes. Future research should focus on ensuring that ACS therapy be given at the most beneficial time for the fetus to optimize its outcome in the event of a preterm birth. Continued research is needed to answer questions on the long-term effects of exposure to ACS therapy, single and multiple, on later neurobehavioral function, disabilities, mental disorders, and any susceptibility to metabolic and cardiovascular disease.

## Electronic supplementary material

Additional file 1: Table S1: Initial perinatal/neonatal outcomes for all infants, those followed, and those not followed to 5 years of age. (DOCX 33 KB)

## Abbreviations

***MACS***: Multiple Courses of Antenatal Corticosteroids for Preterm Birth Study
***MACS-5***: Multiple Courses of Antenatal Corticosteroids for Preterm Birth Study at 5 years of Age
ACS: Antenatal corticosteroids
OR: Odds ratio
CI: Confidence intervals
CIHR: Canadian Institutes of Health Research
SD: Standard deviation
RDS: Respiratory distress syndrome
BPD: Bronchopulmonary dysplasia
IVH: Intraventricular hemorrhage
PVL: Periventricular leukomalacia
NEC: Necrotising enterocolitis
PMR: Perinatal mortality rate
GMFCS: Gross Motor Function Classification System
BRIEF-P: Behavior Rating Inventory of Executive Function- Preschool version (BRIEF-P)
CBCL-1½-5: Child Behavior Checklist-1½-5.

## References

[1] Murphy KE, Hannah ME, Willan AR, Hewson SA, Ohlsson A, Kelly EN, Matthews SG, Saigal S, Asztalos E, Ross S, Delisle MF, Amankwah K, Guselle P, Gafni A, Lee SK, Armson BA, MACS Collaborative Group (2008). Multiple courses of antenatal corticosteroids for preterm birth (MACS): a randomised controlled trial. Lancet.

[2] Asztalos EV, Murphy K, Hannah M, Willan A, Ohlsson A, Kelly E, Saigal S, Ross S, Delisle M-F, Amankwah K, Guselle P, Gafni A, Lee S, Armson BA, Sananes R, Tomat L, Matthews S (2010). Multiple Courses of Antenatal Corticosteroids for Preterm Birth Study (MACS): 2-year outcomes. Pediatrics.

[3] Asztalos EV, Murphy KE, Willan AR, Matthews SG, Ohlsson A, Saigal S, Armson BA, Kelly EN, Delisle MF, Gafni A, Lee SK, Sananes R, Rovet J, Guselle P, Amankwah K, Saleem M, Sanchez J, MACS-5 Collaborative Group (2013). Multiple Courses of Antenatal Corticosteroids for Preterm Birth Study: Outcomes in Children at 5 years of age (*MACS-5*). JAMA Pediatr.

[4] Palisano R, Rosenbaum P, Walter S, Russell D, Wood E, Galuppi B (1997). Development and reliability of a system to classify gross motor function in children with cerebral palsy. Dev Med Child Neurol.

[5] Gioia GA, Isquith PK, Guy PK, Kenworthy L (2000). Behavior Rating Inventory of Executive Function (BRIEF).

[6] Achenbach TM (2002). Manual for the Child Behavior Checklist- 1½-5 years.

[7] Schmidt B, Whyte RK, Asztalos E, Moddeman D, Poets C, Rabi Y, Solimano A, Roberts RS, for the Canadian Oxygen Trial (COT) Group (2013). Effects of targeting higher vs. lowerarterial oxygen saturations on death or disability in extremely preterm infants: a randomized clinical trial. JAMA.

[8] Schmidt B, Davis P, Moddeman D, Ohlsson A, Roberts RS, Saigal S, Solimano A, Vincer M, Wright LL (2001). Trial Of Indomethacin Prophylaxis in Preterms Investigators. Long-term effects of indomethacin prophylaxis in extremely-low-birth-weightinfants. N Engl J Med.

[9] Whyte RK, Kirpalani H, Asztalos EV, Andersen C, Blajchman M, Heddle N, LaCorte M, Robertson CMT, Clarke MC, Vincer MJ, Doyle LW, Roberts RS, and for the PINTOS Study Group (2009). Neurodevelopmental outcome of extremely low birth weight infants assigned to restrictive or liberal haemoglobin thresholds for blood transfusion. Pediatrics.

[10] Schmidt B, Roberts RS, Davis P, Doyle LW, Barrington KJ, Ohlsson A, Tin W, Caffeine for Apnea of Prematurity Trial Group (2006). Caffeine therapy for apnea of prematurity. N Engl J Med.

[11] Steer P, Flint C (1999). ABC of Labour care: preterm labour and premature rupture of membranes. Br Med J.

[12] Davis EP, Waffarn F, Uy C, Hobel CJ, Glynn LM, Sandman CA (2009). Effect of prenatal glucocorticoid treatment on size at birth among infants born at term gestation. J Perinatol.

[13] Waffarn F, Davis EP (2012). Effects of antenatal corticosteroids on the hypothalamic-pituitary-adrenocortical axis of the fetus and newborn: experimental findings and clinical considerations. Amer J Obstet Gynecol.

[14] Alexander N, Rosenlöcher F, Stalder T, Linke J, Distler W, Morgner J, Kirschbaum C (2012). Impact of antenatal synthetic glucocorticoid exposure on endocrine stress reactivity in term-born children. J Clin Endocrin Metab.

[15] Dunlop SA, Archer MA, Quinlivan JA, Beazley LD, Newnham JP (1997). Repeated prenatal corticosteroids delay myelination in the ovine central nervous system. J Matern Fetal Med.

[16] Quinlivan JA, Beazley LD, Evans SF, Newnham JP, Dunlop SA (2000). Retinal maturation is delayed by repeated, but not single, maternal injections of betamethasone in sheep eye. Eye.

[17] Church MW, Adams BR, Anumba JI, Jackson DA, Kruger ML, Jen KLC (2012). Repeated antenatal corticosteroid treatments adversely affect neural transmission time and auditory thresholds in laboratory rats. Neurotxicology and Teratology.

[CR18] The pre-publication history for this paper can be accessed here: http://www.biomedcentral.com/1471-2393/14/272/prepub

