# Supplementary material for: Association between gestational age at birth, antenatal corticosteroids, and outcomes at 5 years: multiple courses of antenatal corticosteroids for preterm birth study at 5 years of age (MACS-5)
Source: BMC Pregnancy Childbirth. 2014 Aug 13;14:272. doi: 10.1186/1471-2393-14-272 (PMC4261573; doi:10.1186/1471-2393-14-272)
Supplement: Supplementary file 1 — Additional file 1: Table S1: Initial perinatal/neonatal outcomes for all infants, those followed, and those not followed to 5 years of age. (DOCX 33 KB) [file 12884_2014_1242_MOESM1_ESM.docx]

**Supplementary Table 1 Initial perinatal/neonatal outcomes for all infants, those followed, and those not followed**

**to 5 years of age**

|  | ***MACS* Infants in Initial Trial** | | ***MACS-5* Infants followed** | | ***MACS-5* Infants not followed** | |
| --- | --- | --- | --- | --- | --- | --- |
|  | **Multiple ACS^a^ Group**  **No of Infants = 1164^b^** | **Single ACS Group**  **No of Infants = 1140** | **Multiple ACS Group**  **No of Infants = 873^a^** | **Single ACS Group**  **No of Infants = 855** | **Multiple ACS Group**  **No of Infants= 292** | **Single ACS Group**  **No of Infants= 285** |
| Mean birth weight grams (SD) | 2171 (793) | 2282 (787) | 2148 (798) | 2262 (797) | 2240 (777) | 2343 (754) |
| Birth weight < 10^th^ centile for gestational age | 196 (16.8%) | 158 (13.9%) | 146 (16.7%) | 124 (14.5%) | 50 (17.1%) | 34 (11.9%) |
| Birth weight < 3rd centile for gestational age | 64 (5.5%) | 59 (5.2%) | 47 (5.4%) | 49 (5.7%) | 17 (5.8%) | 10 (3.5%) |
| Death or serious neonatal morbidity: composite primary outcome (one or more of death, RDS^c^, BPD^d^, IVH^e^ [grade III/IV], cystic PVL^f^, NEC^g^) | 150 (12.9%) | 143 (12.5%) | 119 (13.6%) | 117 (13.7%) | 31 (10.6%) | 26 (9.1%) |
| Stillbirth or neonatal death ≤ 28 days after birth or prior to discharge whichever was later | 43 (3.7%) | 40 (3.5%) | 40 (4.6%) | 40 (4.7%) | 3 (1.0%) | 0 |
| Surviving Infants | 1121 | 1100 | 833 | 815 | 289 | 285 |
| Serious neonatal morbidity (one or more of RDS, BPD, IVH [grade III/IV], cystic PVL, NEC) | 107/1121 (9.6%) | 103/1100 (9.4%) | 79/833 (9.5%) | 77/815 (9.5%) | 28 (9.7%) | 26 (9.1%) |
| RDS^c^ | 87/1121 (7.8%) | 77/1100 (7.0%) | 65/833 (7.8%) | 60/815 (7.4%) | 22/28 (7.6%) | 17/285 (6.0%) |
| BPD^d^ | 19/1121 (1.7%) | 11/1100 (1.0%) | 16/833 (1.9%) | 8/815 (1.0%) | 3/28 (1.0%) | 3/26 (1.1%) |
| IVH^e^ [grade III/IV] | 6/1121 (0.5%) | 9/1100 (0.8%) | 2/833 (0.2%) | 4/815 (0.5%) | 4/28 (1.4%) | 5/26 (1.8%) |
| Cystic PVL^f^ | 9/1121 (0.8%) | 10/1100 (0.9%) | 7/833 (0.8%) | 7/815 (0.9%) | 2/28 (0.7%) | 3/26 (1.1%) |
| NEC^g^ | 10/1121 (0.9%) | 12/1100 (1.1%) | 8/833 (1.0%) | 7/815 (0.9%) | 2/28 (0.7%) | 5/26 (1.8%) |

^a^ ACS = antenatal corticosteroids;

^b^1 lost to follow-up case found and included at 5 years evaluation; no neonatal data available;

^c^ RDS (respiratory distress syndrome) was defined as requiring assisted ventilation via endotracheal tube and supplemental oxygen both within the first 24 hours of life and for a duration of greater than or equal to 24 hours, and either an x-ray compatible with RDS or surfactant given between the first 2 and 24 hours of life;

^d^ BPD(bronchopulmonary dysplasia) was defined as requiring oxygen at a postnatal gestational age of 36 completed weeks and x-ray compatible with BPD;

^e^ IVH = intraventricular hemorrhage

^f^ cystic PVL(periventricular leukomalacia) was defined as periventricular cystic changes in the white matter, excluding subependymal and choroid plexus cysts;

^g^ NEC(necrotizing enterocolitis) was defined as either perforation of intestine, pneumatosis intestinalis or air in the portal vein.
